# Modified Huo-Luo-Xiao-Ling Dan Suppresses Adjuvant Arthritis by Inhibiting Chemokines and Matrix-Degrading Enzymes

**DOI:** 10.1155/2012/589256

**Published:** 2012-03-07

**Authors:** Siddaraju M. Nanjundaiah, David Y.-W. Lee, Zhongze Ma, Harry H. S. Fong, Lixing Lao, Brian M. Berman, Kamal D. Moudgil

**Affiliations:** ^1^Department of Microbiology and Immunology, University of Maryland School of Medicine, HSF-1, Suite 380, 685 West Baltimore Street, Baltimore, MD 21201, USA; ^2^Mailman Research Center, McLean Hospital, Harvard Medical School, Belmont, MA 02478, USA; ^3^Department of Medicinal Chemistry and Pharmacognosy, University of Illinois at Chicago, Chicago, IL 60612, USA; ^4^Center for Integrative Medicine, University of Maryland School of Medicine, East Hall, 520 W. Lombard Street, Baltimore, MD 21201, USA

## Abstract

Rheumatoid arthritis (RA) is a chronic inflammatory disease affecting the joints that can lead to deformities and disability. The prolonged use of conventionally used drugs is associated with severe adverse reactions. Therefore, safer and less expensive therapeutic products are continually being sought. Huo-Luo-Xiao-Ling dan (HLXL), a traditional Chinese herbal mixture, and its modified versions possess anti-arthritic activity. In this paper, we examined the influence of modified HLXL on two of the key mediators of arthritic inflammation and tissue damage, namely, chemokines and matrix-metalloproteinases (MMPs) in the rat adjuvant-induced arthritis (AA) model of RA. We treated arthritic Lewis rats with HLXL (2.3 g/kg) by daily gavage beginning at the onset of AA. The control rats received the vehicle. At the peak phase of AA, rats were sacrificed and their draining lymph node cells (LNC) and spleen adherent cells (SAC) were tested. The HLXL-treated rats showed a significant reduction in the levels of chemokines (RANTES, MCP-1, MIP-1**α**, and GRO/KC), MMPs (MMP 2 and 9), as well as cytokines (IL-6 and IL-17) that induce them, compared to the control vehicle-treated rats. Thus, HLXL controls arthritis in part by suppressing the mediators of immune pathology, and it might offer a promising alternative/adjunct treatment for RA.

## 1. Introduction

Rheumatoid arthritis (RA) is a chronic debilitating autoimmune disease that affects over 1 percent of the population worldwide [1]. The commonly used drugs such as nonsteroidal anti-inflammatory drugs (NSAIDS) and biologics (e.g., antitumor necrosis-*α* antibody) are effective in alleviating the symptoms of the disease. However, the prolonged use of these drugs is associated with severe adverse reactions [2, 3]. In addition, these drugs are expensive, and not all patients respond well to them. In view of these limitations, it is essential to continue the search for safer and less expensive alternatives to the conventionally used drugs [4, 5]. Natural plant products represent a promising group of therapeutic agents for arthritis. However, one of the major concerns in seriously considering these products for therapeutic purposes is that the mechanisms of action of many of them are poorly defined, if at all. 

RA primarily targets the joints, and is characterized by inflammatory synovitis mediated by leukocytes and the proinflammatory cytokines secreted by them [1, 6]. The migration of leukocytes from the peripheral blood into the joints is directed by chemotactic cytokines (chemokines) [7]. Furthermore, severe arthritis is associated with cartilage and bone damage, which is mediated in part by the matrix-degrading enzymes, matrix metalloproteinases (MMPs) [8, 9]. Therefore, chemokines and MMPs are attractive targets for the treatment of arthritis [10, 11].

Chemokines are small, biologically active molecules that attract specific populations of inflammatory cells and regulate their trafficking to the site of inflammation. Among the chemokines that play an important role in inflammation, including RA, are regulated upon activation, normal T cell expressed, and secreted (RANTES), also known as chemokine C-C motif ligand 5 (CCL5); monocyte chemotactic protein-1 (MCP-1), or CCL2; macrophage inflammatory protein-1*α* (MIP-1*α*), or CCL3; and growth regulated oncogene-keratinocyte chemoattractant (GRO/KC), or chemokine C-X-C motif ligand 1 (CXCL1) [12–15]. The chemokines interact with their receptors which belong to seven-transmembrane G-protein-coupled molecules. Henceforth, for simplicity we will refer to the 4 chemokines tested in this study by their common names (RANTES, MCP-1, MIP-1*α*, and GRO/KC).

MMPs are a group of zinc-dependent endopeptidases that can degrade the extracellular matrix (ECM). Among the MMPs, MMP-2 (gelatinase A) and MMP-9 (gelatinase B) are especially important in collagen degradation [16]. The expression/activity of MMPs can be modulated by the proinflammatory cytokines interlukin-6 (IL-6) and IL-17 that are expressed in the synovial tissue and synovial fluid in the joints of RA patients [8, 17].

Huo-luo-xiao-ling dan (HLXL), a traditional Chinese herbal formula, and its modified versions have been used in folk medicine over centuries to treat inflammatory arthritis or joint pain, referred to as the “*Bi syndrome*” [18, 19]. The use of a combination of multiple herbs is designed to exploit the additive or synergistic activities of individual herbs, as well as to balance or neutralize the toxic effects of certain herbal components by others in the mixture [20]. In our previous studies in animal models of arthritis, we have shown that oral administration of modified HLXL, a well-characterized herbal mixture [21–23], to rats can attenuate inflammatory paw edema as well as the swelling and pain associated with clinical arthritis. In addition, HLXL modulates the balance of pro- versus anti-inflammatory cytokines [24]. In this study, we examined the effect of HLXL on specific chemokines and MMPs, which play an important role in the pathogenesis of AA [25]. As the expression/activity of certain chemokines as well as MMPs is influenced significantly by the proinflammatory cytokines (IL-17 and IL-6), we also tested the effect of HLXL on these two cytokines. We observed that HLXL treatment of arthritic rats attenuates the progression of AA by inhibiting chemokines (RANTES, MCP-1, MIP-1*α*, and GRO/KC), MMPs (MMP-2 and MMP-9), and cytokines (IL-6 and IL-17). To the best of our knowledge, this is the first report describing these HLXL-induced changes in arthritis.

## 2. Materials and Methods

### 2.1. Animals

Five- to six-week-old male Lewis (LEW/Hsd) (RT.1^l^) rats were purchased from Harlan Sprague-Dawley (HSD) (Indianapolis, IN, USA) and then maintained in the animal care facility of the University of Maryland School of Medicine, Baltimore. All experimental procedures performed on these rats were in accordance with the guidelines of the Institutional Animal Care and Use Committee (IACUC).

### 2.2. Composition and Characteristics of HLXL

The traditional Huo-Luo-Xiao-Ling Dan (HLXL) consists of four herbs, namely, Danggui (*Angelica sinensis* (Oliv.) Diels), Danshen (*Salvia miltiorrhiza* Bge.), Ruxiang (*Boswellia carterii* Birdw.), and Moyao (*Commiphora myrrha *Engl.). We have previously reported [21–23] the rationale and the nature of modification of this original formula, and the modified HLXL is a defined mixture of 11 herbs, namely, Ruxiang (*Boswellia carterii* Birdw.), Qianghuo (*Notopterygium incisum* Ting ex H.T. Chang), Danggui (*Angelica sinensis* (Oliv.) Diels), Baishao (*Paeonia lactiflora* Pall.), Gancao (*Glycyrrhiza uralensis* Fisch.), Yanhusuo (*Corydalis yanhusuo* W.T. Wang.), Danshen (*Salvia miltiorrhiza* Bge.), Chuanxiong (*Ligusticum chuanxiong* S.H. Qiu.), Qin jiao (*Gentiana macrophylla* Pall.), Guizhi (*Cinnamomum cassia* Presl.), and Duhuo (*Angelica pubescens* Maxim). The compounds isolated from HLXL include steroids, terpenes, alkaloids, flavonoids, glycosides, and acids [21]. The methods for the preparation of HLXL, for the characteristics of its component herbs, and for the assessment of its toxicity have been described in detail elsewhere [22, 23]. As in our earlier studies [23, 24] the batch of HLXL tested in this study was thoroughly characterized by HPLC fingerprinting, which were characterized by the peak shapes, numbers, intensities, and retention times of all individual compounds (data not shown). Moreover, the marker compounds, swertiamarin (from *Gentiana macrophylla*), osthole (*Angelica pubescens*), paeoniflorin (*Paeonia lactiflora*), iso-imperatorin (*Notopterygium incisum*), liquiritin (*Glycyrrhiza uralensis*), columbianadin (*Angelica pubescens*), liquiritigenin (*Glycyrrhiza uralensis*), falcarindiol (*Notopterygium incisum*), angelol B (*Angelica pubescens*), cryptotanshinone (*Salvia miltior*rhiza), bergapten (*Angelica pubescens*), tanshinone IIA (*Salvia miltiorrhiza*), senkyunolide A (*Ligusticum chuanxiong*), ostruthin (*Notopterygium incisum*), phenethyl transferulate (*N. incisum*), and anhydronotoptol (*N. incisum*), served as references for quality control purposes. In a recent study [21], we reported that some of the above-mentioned herbs other than the 4 traditionally used in classical HLXL, possessed compounds that served as ligands for the enzyme cyclo-oxygenase 2 (COX-2). This further reinforces in part the rationale for the modification of the original HLXL formula.

### 2.3. Treatment of Arthritic Rats with HLXL

Lewis rats were immunized subcutaneously (s.c.) with 1 mg/rat heat-killed *M. tuberculosis* H37Ra (Mtb) (Difco, Detroit, MI) in 200 *μ*L of mineral oil (Sigma-Aldrich) at the base of the tail. Following the onset of arthritis, these rats were randomly divided into two groups (experimental and control). HLXL was finely powdered and suspended in 200 mL of water using a pestle and mortar, and it was fed (2.3 g/kg) to the experimental group of rats using a gavage needle (FNC-16-3, Kant Scientific Corporation, Torrington, CT, US) beginning on the day of onset of arthritis (d 10) and then continued up to the peak phase of AA (d 18). On the corresponding days, the control group of rats received water (the vehicle) by gavage. All rats were examined and graded regularly for the severity of arthritis as described earlier [24, 26]. The test samples were collected from rats when the disease reached the peak phase (d 18) in controls.

### 2.4. Preparation and Antigenic Restimulation of Lymph Node Cells (LNC)

Rats treated either with HLXL or with vehicle (water) only were euthanized at the peak phase of AA (d 18) and their draining lymph nodes (para-aortic, inguinal and popliteal) were harvested [26]. A single cell suspension of LNC was prepared in HL-1 serum free medium (Ventrex Laboratories, Portland, ME) supplemented with 2 mM L-glutamine, 100 U/mL penicillin G sodium, and 100 *μ*g/mL streptomycin sulfate. In each experiment, LNC from 3 rats were pooled for testing. These LNC were cultured (8 × 10^6^ cells/well) in a 6-well plate (Corning Incorporated Corning, NY) for 24 h at 37°C in an atmosphere of 95% air and 5% CO_2._ Sonicated Mtb (10 *μ*g/mL) was used for restimulation of LNC. Keyhole limpet hemocyanin (KLH) was used as the control antigen. Total RNA was prepared from these LNC for testing by quantitative real-time polymerase chain reaction (qRT-PCR) as described below. Alternatively, the LNC culture supernatant was harvested for testing by a Multiplex assay as elaborated below.

### 2.5. Spleen Adherent Cells (SAC) and Their Stimulation with Sonicated Mtb

Spleens were harvested from experimental/control rats (*n* = 3 each) at the peak phase of AA (d 18) and a single cell suspension was prepared as described above for LNC. These spleen cells were allowed to settle in a 6-well plate at 37°C in RPMI medium supplemented with 5% fetal bovine serum (FBS), 2 mM L glutamine, 100 U/mL penicillin G sodium, and 100 *μ*g/mL streptomycin sulfate. After 90 min, nonadherent cells were removed by washing with HBSS (Invitrogen, Frederick, MD), yielding the SAC [27]. These SAC (1.5–2.0 × 10^6^ cells per well) were restimulated for 6 h with sonicated Mtb (10 *μ*g/mL). KLH served as the control antigen. Thereafter, culture supernatants were collected for testing in Multiplex assay. Alternatively, total RNA was prepared from these SAC as described below.

### 2.6. Determination of Matrix Metalloproteinase (MMP)/Cytokine mRNA Expression by qRT-PCR

RNA was isolated from LNC and SAC using TRIzol reagent (Invitrogen, Carlsbad, CA) and then cDNA was prepared from RNA using iScript cDNA synthesis kit (Bio-Rad laboratories, Hercules, CA). The resultant cDNA was amplified in an ABI Prism 7900HT cycler (Applied Biosystems) by qRT-PCR using SYBR Green PCR Master Mix (Applied Biosystems) and the appropriate primers for MMP-2, MMP-9, IL-6, and IL-17. These primers were synthesized at the Biopolymer Core Facility, University of Maryland, Baltimore. The mRNA levels of the genes of interest were normalized to the hypoxanthine-guanine phosphoribosyltransferase (HPRT) gene, and the relative gene expression levels were expressed as “fold increase” [26].

### 2.7. Determination of Chemokine/Cytokine Protein Expression by Multiplex Suspension Bead Array Immunoassay

Multiplex assays were performed at the Cytokine Core Facility (University of Maryland, Baltimore) using the Luminex 100 analyzer (Luminex Corp., Austin, TX) and the level of cytokines (as protein) in 24 h culture supernatant of LNC and SAC were measured. The Rat chemokine 4-plex kit (Millipore) was used to measure RANTES, MCP-1, MIP-1*α*, and GRO/KC and the Rat cytokine 2-plex kit was used to measure IL-6 and IL-17.

### 2.8. Measurement of MMP Activity Using a Zymogram Assay

The MMP activity in the culture supernatants of LNC and SAC stimulated with or without Mtb was determined using a zymogram assay as described previously [26, 28]. Briefly, culture supernatant was loaded onto a gelatin-coated, pre-casted polyacrylamide gel (Bio-Rad). Electrophoresis was carried out under SDS-nonreducing conditions at constant voltage. The gel was incubated with 2.5% Triton X-100 at room temperature for 1-2 h to remove SDS. The gel was then washed 3-4 times with water to remove Triton X-100 and incubated overnight at 37°C in a developing buffer (Tris-HCl, pH 7.4) containing 5 mM CaCl_2_, 0.2 M NaCl, and 0.02% Brij 35. Thereafter, the gel was stained with Coomassie Brilliant Blue R-250. Standard MMP-2 and MMP-9 (Sigma) were used as positive controls. The MMP activity was visualized and scanned after destaining. Thereafter, the intensity of the bands was quantitated by densitometry using Image J software.

### 2.9. Statistical Analysis

The data were expressed as mean ± SEM. Student's *t*-test and ANOVA were used to assess the significance of differences using GraphPad Prism version 4.0. A *P* value of <0.05 was considered significant.

## 3. Results

As reported earlier [24], we observed in this study that HLXL treatment of arthritic Lewis rats reduced the severity of AA. The mean arthritic score on d 18 (peak phase of AA), was 2.2 for HLXL-treated group compared to 4.6 for the control water-treated group, and this difference was statistically significant (*P* < 0.02). We then determined the effect of HLXL on specific chemokines, MMPs and cytokines on d 18 of arthritis and compared the results with those obtained from the control rats. The results are presented below.

### 3.1. HLXL Treatment Downmodulates Chemokine Production in Arthritic Lewis Rats

**Figure 1:**
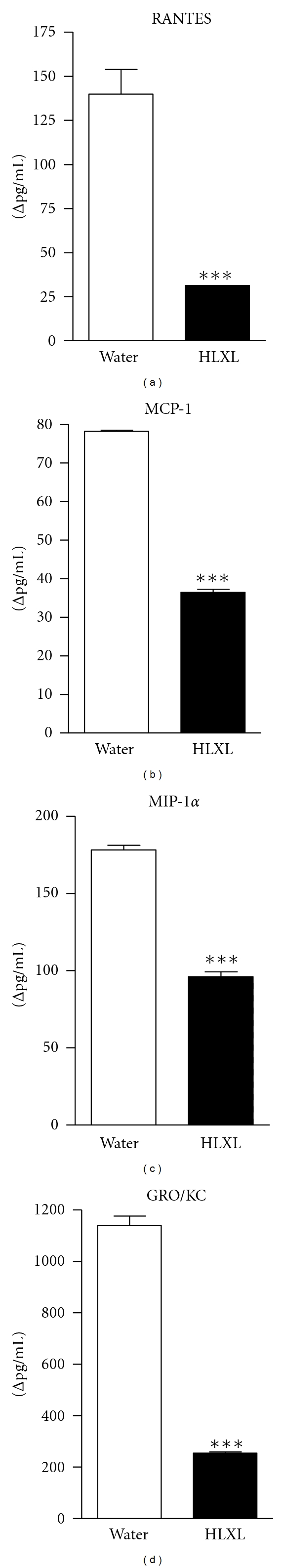
Suppression of chemokines in HLXL-treated arthritic rats. Lymph node cells (LNC) harvested at the peak phase of the disease (d 18) from arthritic rats fed with HLXL or the vehicle (water) were cultured in vitro for 24 h in the presence or absence of sonicated Mtb. Thereafter, the culture supernatants were harvested and tested for chemokines: RANTES (a), MCP-1 (b), MIP-1*α* (c), and GRO/KC (d). The results were expressed as Δpg/mL. Representative results of one of two independent experiments are shown (***P < 0.005) (Mtb = heat-killed *M. tuberculosis *H37Ra).

Chemokines and their receptors coordinate the movement of cells of the immune system and direct these cells to the site of inflammation. The antigen-draining lymph nodes are the site of initial cellular activation and interactions. In this context, we tested the effect of HLXL treatment on chemokines produced by the draining LNC. Specifically, we tested for RANTES, MCP-1, MIP-1*α*, and GRO/KC. These chemokines were measured in culture supernatants of the draining LNC restimulated with sonicated Mtb for 24 h (Figure 1). We observed a 4.4-fold decrease in RANTES in HLXL-treated group, which was highly significant (*P* < 0.001) when compared to the control group. There was a 2.1- and 1.6-fold decrease in MCP-1 (*P* < 0.00001) and MIP-1*α* (*P* < 0.001), respectively after HLXL treatment. GRO/KC showed a marked downregulation in HLXL-treated group with a 4.5-fold decrease (*P* < 0.001) compared to the control group.

### 3.2. HLXL Suppresses MMP-9 and MMP-2 Activity in Arthritic Rats

**Figure 2:**
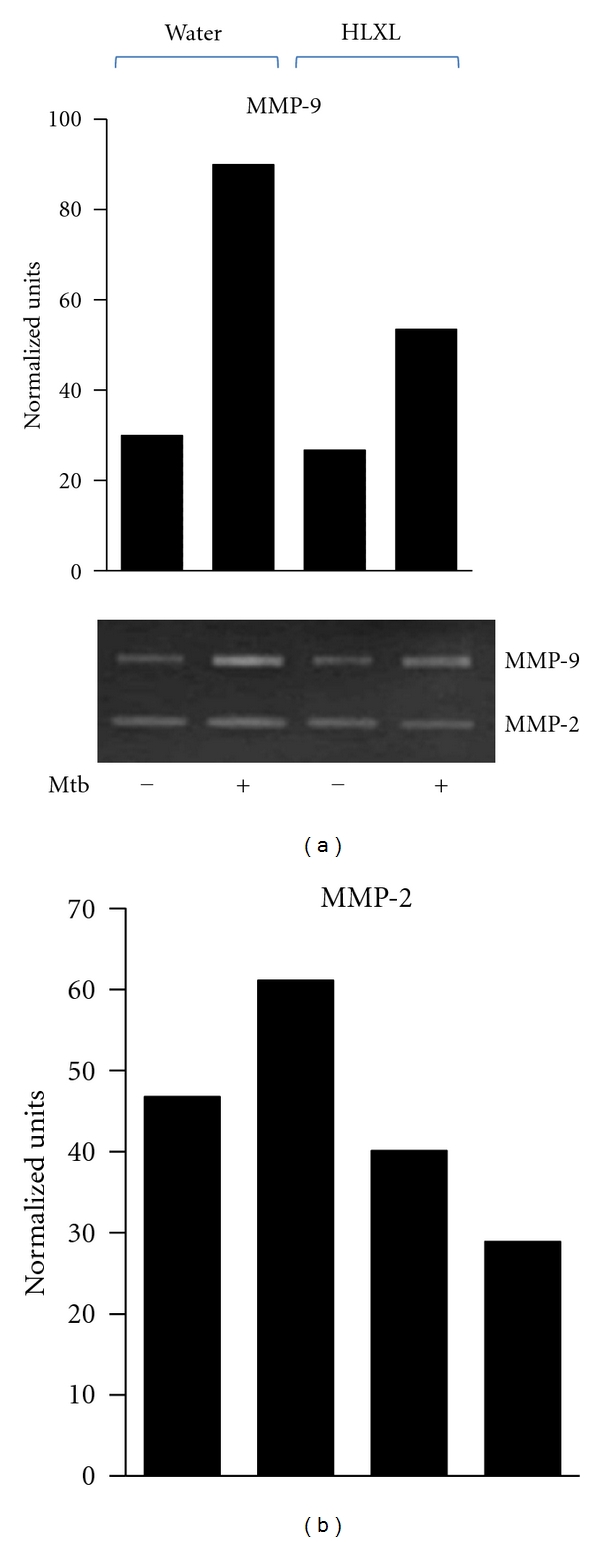
The effect of HLXL on MMP activity in arthritic rats. Spleen adherent cells (SACs) harvested from rats fed with HLXL or the vehicle (water) were stimulated for 24 h with sonicated Mtb. The supernatants obtained from these cultured cells were analyzed for MMP-9 (a) and MMP-2 (b) activity using a zymogram assay. The results are representative of two independent experiments (Mtb = heat-killed *M. tuberculosis *H37Ra).

MMPs mediate the degradation of extracellular matrix macromolecules, and they play an important role in cartilage and bone damage in arthritic joints. Therefore, to further understand the mechanisms underlying the arthritis-protective effect of HLXL, we evaluated the mRNA expression and enzyme activity of MMPs in SAC that were harvested from HLXL-treated or control rats and then restimulated with sonicated Mtb for 24 h. Treatment with HLXL caused a 1.7-fold decrease in the expression of MMP-9 but no significant change in MMP-2 mRNA expression (see Supplementary Figure  1 in Supplementary material available online at doi: 10.1155/2012/589256). Furthermore, there was 2.3-fold suppression in the activity of MMP-9 and a 2.7-fold decrease in MMP-2 activity in HLXL-treated group compared to the controls (Figure 2).

### 3.3. HLXL Treatment Inhibits Antigen-Induced Pro-Inflammatory Cytokine Response in Arthritic Rats

**Figure 3:**
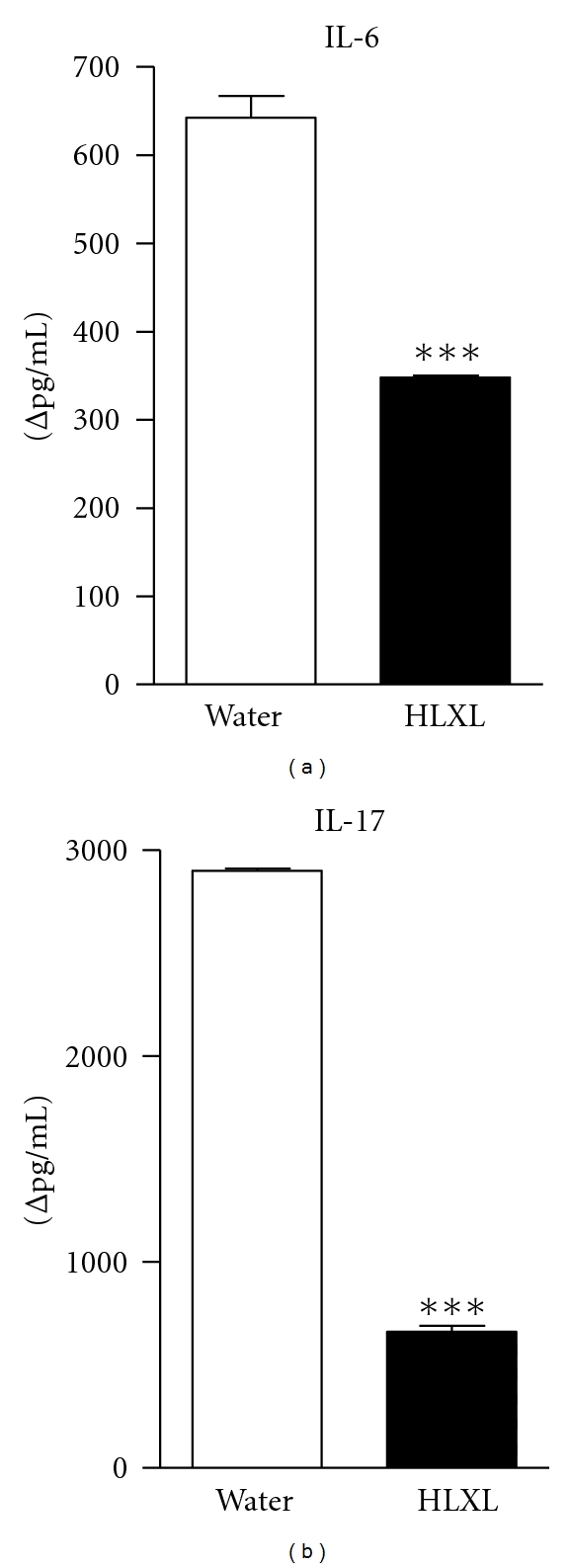
Inhibition of proinflammatory cytokines in HLXL-treated arthritic rats. Lymph node cells (LNCs) harvested on d 18 (peak phase of arthritis) from HLXL-fed or water-fed rats were cultured for 24 h with or without sonicated Mtb. The culture supernatants were tested for IL-6 (a) and IL-17 (b) using a multiplex assay. The results were expressed as Δpg/mL. The results are representative of two independent experiments. (Mtb = heat-killed *M. tuberculosis *H37Ra).

IL-6 and IL-17 are proinflammatory cytokines that have a significant effect on different chemokines and MMPs involved in the arthritogenic processes. Therefore, we tested the levels of these two cytokines (as proteins) in the draining LNC that were harvested from HLXL-treated and control arthritic rats and then restimulated in vitro for 24 h with sonicated Mtb. There was a significant decrease in the level of IL-6 (*P* < 0.0002) and IL-17 (*P* < 0.0001) in HLXL-treated rats compared with water-treated rats (Figures 3(a) and 3(b)). HLXL-treated group showed a 1.8-fold decrease in IL-6 and a 4.3-fold decrease in IL-17 compared to the controls. Furthermore, in SACs stimulated with sonicated Mtb, there was a 14 fold decrease in IL-6 and a 3.4 fold reduction in IL-17 in HLXL-treated rats compared to vehicle-treated rats (see Supplementary Figure  2). Thus, HLXL inhibited cytokine production in LNC as well as SAC, indicating a systemic effect.

## 4. Discussion

In this study based on the rat AA model of human RA, we observed that HLXL-treated rats had significantly reduced levels of chemokines (RANTES, MCP-1, MIP-1*α*, and GRO/KC) as well as matrix-degrading enzymes, MMP2 and MMP9. Also reduced were the levels of the proinflammatory cytokines IL-6 and IL-17.

### 4.1. The Involvement of Chemokines in the Pathogenesis of Arthritis and the Therapeutic Targeting of Chemokines

 High levels of RANTES are found in the synovial fluid and serum obtained from patients with RA, and it promotes the migration of mononuclear cells (including lymphocytes) from blood vessels and synovial fluid into the synovium [12]. MCP-1 is chemotactic for monocytes and T lymphocytes [15] whereas GRO-*α* is a chemoattractant for neutrophils in RA [13]. Elevated levels of MIP-1*α* are found in sera and synovial fluid of RA patients suggesting that MIP-1*α* plays an important role in the progression of the disease [14]. Initial clinical trials on chemokine blockade in patients with RA suggest that targeting the chemokine and chemokine receptor family might provide a promising therapeutic approach for this deliberating disease [10]. In this context, the results of our study showing that the above-mentioned 4 chemokines are suppressed in HLXL-treated rats compared to the controls are of high significance.

 In the AA model, high levels of RANTES are found in the peripheral blood at the onset of the disease, whereas MCP-1 is first detected in the synovial tissue and later detected in the peripheral blood on day 18 (peak phase of AA), when joint inflammation is already very active [25]. These results suggest that RANTES is essential for the initiation of the disease, whereas MCP-1 follows the disease onset. Blocking the receptor for MCP-1 before the onset of arthritis in AA affords protection against the disease [29]. In our study reported here, we observed a positive correlation between arthritic scores and MCP-1 levels, with both being reduced after HLXL treatment. Furthermore, our findings of suppression of MCP-1 and RANTES after HLXL treatment are supported by those of another study showing reduced serum levels of MCP-1 and RANTES in RA patients following treatment with formulations of dried encapsulated juice concentrate [30]. Similarly, these chemokines can be suppressed by green tea extract [31]. In regard to GRO/KC, it was shown in the CIA model that this chemokine is elevated in the synovial fluid of arthritic rats and that its levels are reduced following suppression of arthritis by an inhibitor of spleen tyrosine kinase [32]. The results of our study in the AA model showed significant suppression of GRO/KC in HLXL-treated rats, which was associated with reduced severity of AA compared to the control rats.

### 4.2. The Induction and Regulation of MMPs by Pro-Inflammatory Cytokines and Chemokines, and the Control of MMP Activity for Therapeutic Purposes

 Macrophages and fibroblasts synthesize and secrete several MMPs that participate in the degradation of ECM components and contribute significantly to the tissue damage in RA [9]. Furthermore, MMP-9 is believed to play a key role in directing the migration of macrophages and neutrophils in RA [8, 33]. Synovial fluid from RA patients contains high levels of MMP-2 and MMP-9 [16]. Therefore, MMP-2 and MMP-9 are attractive targets for the treatment of RA [11]. In this context, our results showing that HLXL decreases MMP-9 and MMP-2 activity in AA are of direct relevance to RA. 

 Besides MMPs, the increased activity of the proinflammatory cytokines such as IL-6 and IL-17 is closely associated with the destruction of cartilage and bone in RA. IL-6 stimulates the production of MMP-2 and MMP-9 [17]. IL-17 also enhances MMP-2 and MMP-9 expression and is synergistic with IL-1*β* and TNF-*α* in inducing the expression of other proinflammatory cytokines and MMPs [8]. We observed that both IL-6 and IL-17 (as proteins) were significantly reduced after HLXL treatment. This is the first report on HLXL-mediated suppression of IL-6 in AA. In addition, our study revealed the inhibitory effect of HLXL on IL-17 at the protein level, validating our earlier finding on the reduction of IL-17 mRNA expression [24]. We suggest that the downregulation of IL-6 and IL-17 had a significant influence, either directly or indirectly, on the MMP activity, which was found to be decreased in HLXL-treated rats. 

 Chemokines also regulate the expression and activity of MMPs. For example, RANTES and MCP-1 activate monocytes to secrete TNF-*α*, and TNF-*α* in turn stimulates MCP-1, which then induces MMP-9 production by monocytes [34]. On the basis of our results, we suggest that HLXL-induced reduction in MCP-1 might have contributed in part to the reduced production of MMP-9.

### 4.3. The Bidirectional Interplay between Pro-Inflammatory Cytokines and Chemokines

 Overproduction of IL-6 is a characteristic feature of RA, and inhibiting IL-6 activity is one of the therapeutic options for treating RA. Other investigators [35] have shown that RANTES and MCP-1 regulate IL-6 production by fibroblast-like synoviocytes in RA. These observations support our results of HLXL-induced concurrent decrease in the above-referenced chemokines as well as IL-6. The other prominent proinflammatory cytokine, IL-17, stimulates the production of multiple chemokines such as MCP-1 and GRO/KC [36]. In our study, we have shown the concurrent downregulation of chemokines and IL-17 in HLXL-treated rats compared to the control rats.

**Figure 4:**
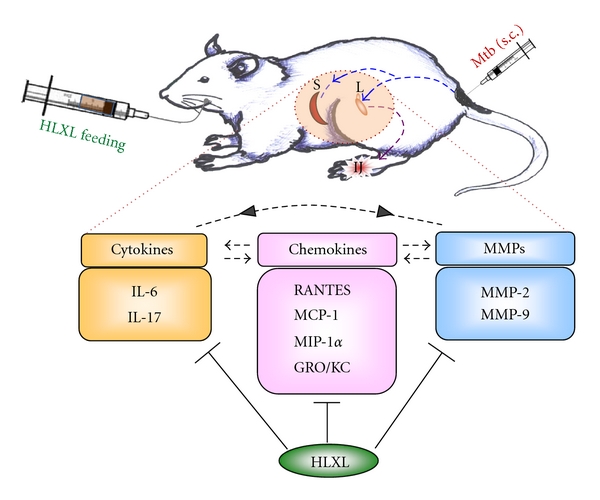
A schematic representation of the diverse mechanisms involved in HLXL-induced suppression of AA. HLXL attenuated arthritis via suppressing the disease-regulating chemokines (regulated upon activation, normal T cell expressed and secreted: RANTES; monocyte chemotactic protein-1: MCP-1; macrophage inflammatory protein-1*α*: MIP-1*α*; growth regulated oncogene-keratinocyte chemoattractant: GRO/KC), matrix metalloproteinases (MMP-2 and MMP-9), and cytokines (IL-6 and IL-17). (S; spleen, L; lymph node, IJ; inflamed joint, Mtb; heat-killed* Mycobacterium tuberculosis *H37Ra).

In conclusion, HLXL acts on different chemokines, MMPs, and cytokines and suppresses their expression, which in turn leads to attenuation of inflammatory arthritis (Figure 4). The precise mechanisms of inhibition of these mediators of inflammation are currently under investigation.
